# Sustained Lung Inflammation Post‐SARS‐CoV‐2 Infection in Mice Is Associated with Increased Pulmonary T Cells

**DOI:** 10.1002/eji.70043

**Published:** 2025-08-24

**Authors:** Sophie Y. Guan, Patricia P. Ogger, Ana Farias, Minerva Garcia Martín, Joy Nakawesi, Olivia Bedard, Candice Baker, Nadia Rosenthal, Cecilia Johansson

**Affiliations:** ^1^ Respiratory Infections Section National Heart and Lung Institute, Imperial College London UK; ^2^ The Jackson Laboratory Bar Harbor Maine USA; ^3^ Cardiac Function Section National Heart and Lung Institute, Imperial College London UK

**Keywords:** immune responses, infectious diseases, inflammation, lung inflammation, T cells

## Abstract

Many SARS‐CoV‐2 patients experience chronic pulmonary symptoms and long‐term inflammation despite viral clearance. While these clinical manifestations have been linked to the dysregulation of the adaptive immune response, the underlying immunopathology remains poorly understood due to a lack of suitable animal models. To investigate long‐term pulmonary consequences of SARS‐CoV‐2 infection, we used a genetic cross of 129 mice and C57BL/6 (B6)‐K18‐*hACE2* transgene mice, a model previously shown to survive infection. 129xB6‐K18‐*hACE2* mice or littermate controls were infected with a low dose (5 × 10^2^ PFU) of ancestral SARS‐CoV‐2. Complete viral clearance and full recovery from weight loss occurred by day 8 post‐infection. However, prolonged inflammation in the lung and airways persisted up to day 28 post‐infection and was associated with the presence of CD4^+^ and CD8^+^ T cells, particularly CD8^+^ effector T cells. This model may therefore prove valuable for further understanding of drivers of long‐term lung inflammation and for testing therapeutic strategies and clinically relevant interventions that can target long‐term pulmonary inflammation following SARS‐CoV‐2 infection.

## Graphical abstract

Low‐dose SARS‐CoV‐2 infection in 129 × C57BL/6‐K18‐*hACE2* mice induces sustained lung inflammation. Despite complete viral clearance and full recovery of body weight by day 8 post‐infection, both CD4^+^ and CD8^+^ T cells remained detectable in the lungs up to day 28 post infection, indicating ongoing immune activity.

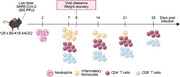

## 1 Introduction

SARS‐CoV‐2, the causative agent of COVID‐19, emerged in late 2019 and rapidly escalated into a global health crisis, causing more than 7 million deaths worldwide [1]. The virus is known for its high transmission rate and spectrum of disease manifestations ranging from asymptomatic infections to severe respiratory distress and death. While progress has been made in understanding the acute phase of SARS‐CoV‐2 infection, including the development of effective vaccines and treatments, the long‐term consequences of the infection present ongoing challenges [2].

Approximately 10%–30% of patients can develop long COVID or post‐acute sequelae of SARS‐CoV‐2 infection (PASC), which can manifest in persistent inflammation affecting multiple organs [3, 4]. While PASC remains a heterogeneous condition, it is often characterised by long‐lasting respiratory symptoms and pulmonary complications [5, 6]. Recently, persistent SARS‐CoV‐2‐specific T cells and increased inflammatory markers were identified in patients with long COVID [7, 8]. While these T cells are important in viral clearance [9] and confer protection by secreting cytokines in mild COVID‐19 [10, 11], they have been shown to contribute to cytokine release syndrome in severe disease [12, 13]. Moreover, during the peak of infection, CD8^+^ T cells within the lungs are linked to inflammatory and fibrotic responses, vascular damage, and adverse clinical outcomes [14].

While it is apparent that SARS‐CoV‐2‐specific T cells play a role in the development of long COVID, their relationship with respiratory symptoms remains to be elucidated. This is due, in part, to the scarcity of suitable mouse models to study the effects of the virus and inflammatory processes. Mouse‐adapted strains of SARS‐CoV‐2 might not fully recapitulate the pathogenesis of SARS‐CoV‐2 in humans [15]. Similarly, adeno‐associated virus (AAV)‐mediated expression of human ACE2 (hACE2) in mice, while informative, predominantly results in mild infection, thus not modelling severe disease states observed in humans.[16, 17] Transgenic expression of hACE2 results in disease severity that more closely reflects severe human infection [18, 19]. Initially, the K18‐*hACE2* mouse model was so severe that mice reached the humane endpoint by around 6 days post infection (d.p.i.).[20, 21] Even lower infection doses, such as 2 × 10^4^ and 2 × 10^3^ PFU or 10^3^ TCID_50_ SARS‐CoV‐2, resulted in 40%–50% of mice reaching the humane endpoint by 7–11 d.p.i. [22, 23, 24]. The severity of disease following low‐dose infection (e.g., 10^2^ PFU) SARS‐CoV‐2 remains controversial, with some studies reporting only 60% survival by 11 d.p.i. [22], while others observe sublethal disease manifestations [19]. Therefore, K18‐*hACE2* mice (C57BL/6) have been crossed with other backgrounds to reduce disease severity and allow the study of longer‐term effects of SARS‐CoV‐2 infections [25].

Here, we describe a long‐term infection model of SARS‐CoV‐2 to study lung inflammation using a genetic cross of 129S1 and C57BL/6‐K18‐*hACE2*. As opposed to the original C57BL/6‐K18‐*hACE2* mice, the 129‐F1 cross is resistant to pathology upon infection with ancestral SARS‐CoV‐2 [25], enabling the investigation of time points later than 6 d.p.i., and particularly the study of long‐term effects. In this mouse model, we report robust immune cell infiltration into the lungs and airways that is sustained after viral clearance and weight recovery up to 28 d.p.i. This long‐term inflammation can be attributed to CD4^+^ and CD8^+^ T cells, providing an effective model to test therapeutic strategies and clinically relevant interventions.

## 2 Results

### 2.1 Exposure to Low Dose SARS‐CoV‐2 Results in Transient Disease in *129xB6‐K18‐hACE2* Mice

**FIGURE 1:**
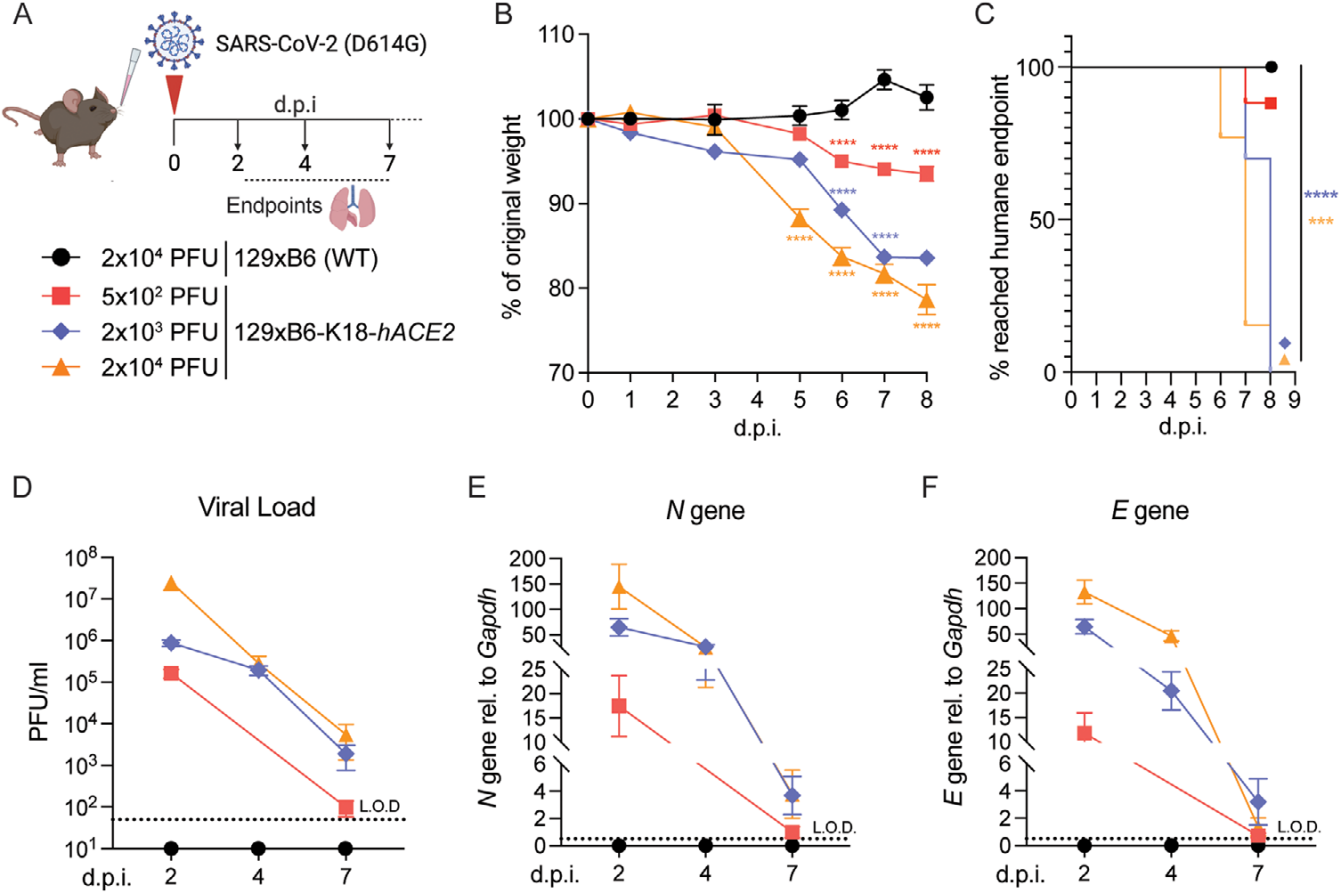
**Low‐dose SARS‐CoV‐2 infection results in symptomatic infection of 129xB6‐K18‐*hACE2* mice**. **(A)** 129xB6‐K18‐*hACE2* and WT mice were intranasally infected with SARS‐CoV‐2 WT (D614G). Lungs and BAL were harvested at 2, 4, and 7 d.p.i. or monitored up to 8 d.p.i. for weight loss. **(B)** Weight loss of 129xB6‐K18‐*hACE2* mice post‐infection with SARS‐CoV‐2. **(C)** Kaplan–Meier analysis of mice reaching humane endpoint infection with SARS‐CoV‐2. **(D)** Viral load measured by plaque assay on Vero cells overexpressing hACE2 and TMPRESS2. **(E)** Expression of SARS‐CoV‐2 *N* gene and **(F)** *E* gene in lung tissue relative to *Gapdh* measured by qRT‐PCR. L.O.D.= Limit of detection. All data are shown as mean ± SEM. In (B–C), 129xB6 (WT) *n* = 10 (two experiments); 129xB6‐K18‐*hACE2* 2 × 10^3^ PFU *n* = 10 (1 experiment), 2 × 10^4^ PFU *n* = 13 (three experiments), 5 × 10^2^ PFU *n* = 61 (5 experiments). In (D–F), *n* = 6–14 per group for 2 × 10^3^ and 2 × 10^4^ PFU doses (two experiments) and *n* = 9–12/group for 5 × 10^2^ PFU (three experiments). Statistical analysis in (B) was performed using two‐tailed Student's *t*‐test to compare each infection dose of 129xB6‐K18‐*hACE2* with 129xB6 (WT) at each post‐infection time point, and log‐rank Mantel–Cox test was performed in (C); *p < 0.05, **p < 0.01, ***p < 0.001, ****p < 0.0001.

In this study, the F1 cross between K18‐*hACE2* mice on a C57BL/6 background and 129S1/SvlmJ mice, hereafter referred to as 129xB6‐K18‐*hACE2* mice, was used. These mice were previously described as “resistant” to SARS‐CoV‐2 infection at titres up to 10^3^ plaque‐forming units (PFU) [25]. Littermates without the *hACE2*‐TG (129xB6; hereafter termed WT) served as controls. To identify a lower, more clinically relevant virus dosage that induces symptomatic but non‐lethal disease, 129xB6‐K18‐*hACE2* mice were infected with 5 × 10^2^, 2 × 10^3^, or 2 × 10^4^ PFU of first‐wave SARS‐CoV‐2 (D614G; Figure 1A). WT mice receiving 2 × 10^4^ PFU SARS‐CoV‐2 remained uninfected and asymptomatic and were used as controls (Figure 1B).

Similar to the original K18‐*hACE2* models [21], 129xB6‐K18‐*hACE2* mice infected with 2 × 10^3^ PFU or 2 × 10^4^ PFU SARS‐CoV‐2 developed severe weight loss of up to 15% or 20%, respectively (Figure 1B), with the majority reaching the humane endpoint by 7–8 d.p.i. (Figure 1C). 129xB6‐K18‐*hACE2* mice inoculated with a lower dose of 5 × 10^2^ PFU SARS‐CoV‐2 exhibited up to 10% of body weight loss and largely did not reach the humane endpoint by 8 d.p.i., showing signs of less severe disease compared with those inoculated with higher doses (Figure 1B,C). High inoculum doses (2 × 10^4^ and 2 × 10^3^ PFU) resulted in high infectious viral titres in the lungs, reaching 10^6^ PFU/ml or more at 2 d.p.i. and persisting at 10^4^ PFU/ml by 7 d.p.i. (Figure 1D). Despite reduced disease severity, 129×B6‐K18‐*hACE2* mice infected with 5 × 10^2^ PFU SARS‐CoV‐2 had high lung viral titres (≥10⁵ PFU/ml) at 2 d.p.i., indicating robust replication (Figure 1D). By 7 d.p.i., the infectious virus was nearly cleared, approaching the detection limit. This pattern was confirmed by qPCR for SARS‐CoV‐2 nucleocapsid (*N*) and envelope (*E*) genes (Figure 1E,F). Thus, SARS‐CoV‐2 low‐dose infection of 129xB6‐K18‐*hACE2* mice induces symptomatic infection, supports early lung viral replication, and allows survival beyond 8 d.p.i.

### 2.2 Sustained Inflammation in the Airways after Viral Clearance

**FIGURE 2:**
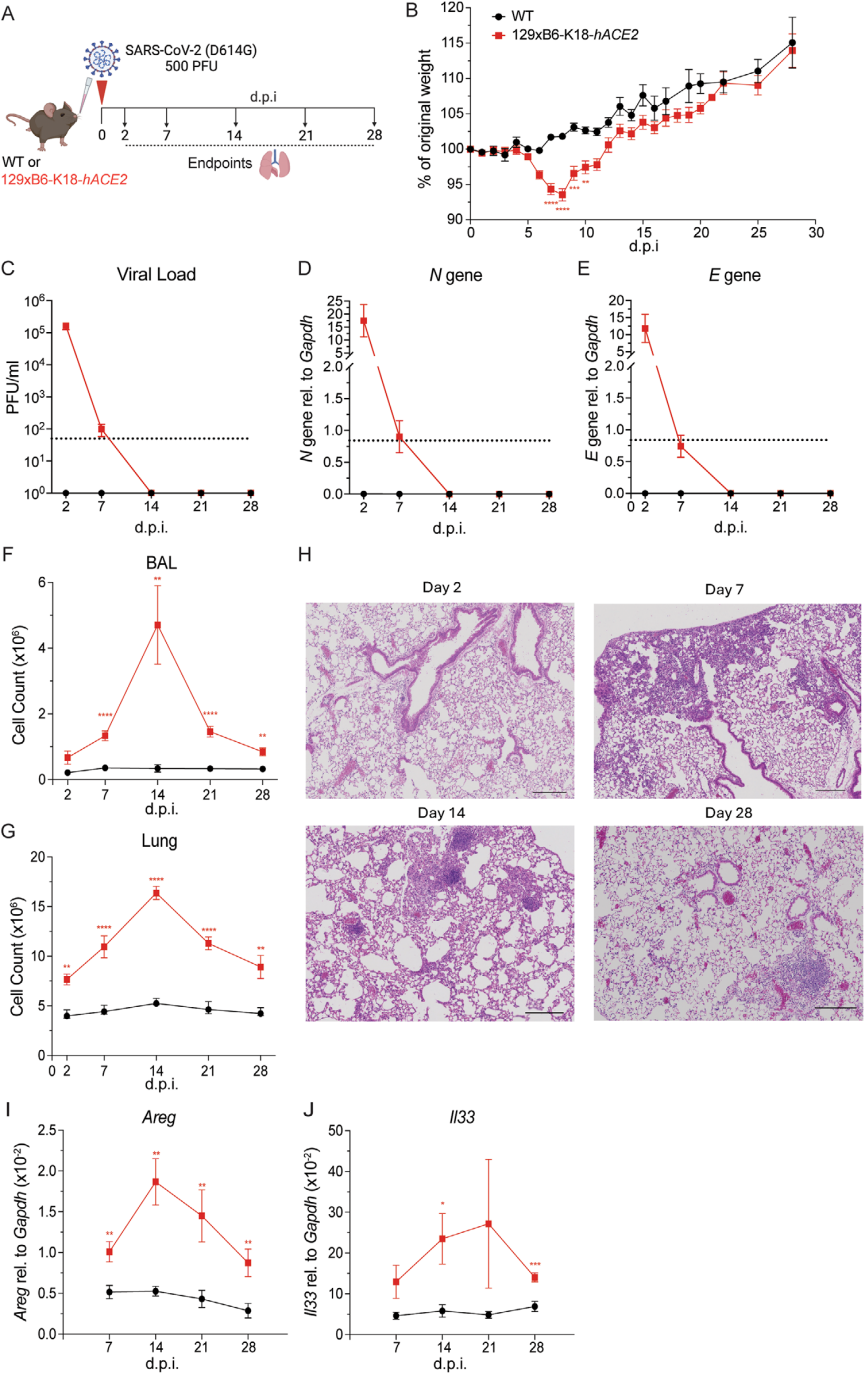
**Persistent inflammation in the airways after weight recovery and viral clearance**. **(A)** 129xB6‐K18‐*hACE2* and WT mice were intranasally infected with SARS‐CoV‐2 (D614G). Lungs and bronchoalveolar lavage (BAL) fluid were harvested at 2, 7, 14, 21, and 28 d.p.i. **(B)** Weight loss (in %) following SARS‐CoV‐2 over 28 days, pooled from five experiments. Mice were culled successively over time for each time point of interest. Viral load measured by **(C)** plaque assay on Vero cells overexpressing hACE2 and TMPRESS2, **(D)** expression of SARS‐CoV‐2 *N* gene and **(E)** *E* gene, quantified via qRT‐PCR. The dotted line represents the limit of detection. **(F, G)** Total cell numbers in the BAL and lung. **(H)** Representative H&E staining of SARS‐CoV‐2 infected lungs in 129xB6‐K18‐*hACE2* mice at 2, 7, 14, and 28 d.p.i. Scale bars = 200 µm. **(I, J)** Gene expression of *Areg* and *Il33* in the lung, relative to *Gapdh*, measured via qRT‐PCR. All data are shown as mean ± SEM. At 7 d.p.i., *n* = 12/group (three experiments); at 2, 14, 21, and 28 d.p.i., *n* = 8–10/group (two experiments). Data up to 8 d.p.i. are also included in Figure 1B–E. Statistical analysis was conducted using two‐tailed Student's *t*‐test within each time point post‐infection. *p < 0.05, **p < 0.01, ***p < 0.001, ****p < 0.0001.

To characterise the long‐term effects of SARS‐CoV‐2 infection in this model, WT and 129xB6‐K18‐*hACE2* mice were intranasally inoculated with 5 × 10^2^ PFU of SARS‐CoV‐2. Weight was monitored until 28 d.p.i., and lung tissue and bronchoalveolar lavage (BAL) fluid were analysed at days 2, 7, 14, 21, and 28 p.i. (Figure 2A). Upon infection, 129xB6‐K18‐*hACE2* mice experienced significant weight loss, peaking at 8 d.p.i., but recovered fully by 12 d.p.i. (Figure 2B). Infectious virus detected in the lungs of 129xB6‐K18‐*hACE2* mice at 2 d.p.i. was completely cleared by 14 d.p.i. (Figure 2C). This pattern was confirmed by gene expression analysis of SARS‐CoV‐2 *N* and *E* genes (Figure 2D,E). Immune cell infiltration in the BAL and lung of infected 129xB6‐K18‐*hACE2* mice, indicative of airway inflammation, peaked at 14 d.p.i. and remained significantly increased compared with WT mice at 28 d.p.i. (Figure 2F,G).

For spatial visualisation of lung inflammation, hematoxylin and eosin (H&E) staining was performed on lung sections at different time points after infection. Infected 129xB6‐K18‐*hACE2* mice showed widespread immune cell infiltration by 14 d.p.i., persisting up to 28 d.p.i. in the perivascular and interstitial spaces (Figure 2H). To further investigate tissue injury, we analysed gene expression of amphiregulin (*Areg*) and *Il33* in lung tissue, which were significantly elevated in infected 129xB6‐K18‐*hACE2* mice, peaking between 14 and 21 d.p.i. and remained upregulated at 28 d.p.i. (Figure 2I,J). Together, these data show that despite weight recovery and virus clearance, 129xB6‐K18‐*hACE2* mice developed extended lung inflammation upon low‐dose SARS‐CoV‐2 infection.

### 2.3 Early Infiltration of Neutrophils and Inflammatory Monocytes

The innate immune system, while crucial for the initial defence against pathogens and the activation of adaptive immunity, may contribute to pulmonary pathology through an overactive inflammatory response [26, 27]. To investigate innate immune responses to low‐dose SARS‐CoV‐2 infection in 129xB6‐K18‐*hACE2* mice, we measured proinflammatory cytokine expression in the airways. IFN‐α protein and gene expression of *Il6, Cxcl1*, *Ccl2*, *Cxcl10*, and *Il10* were significantly higher in the infected 129xB6‐K18‐*hACE2* mice at 2 d.p.i. compared with WT mice, with levels decreasing over time but remaining elevated at 28 d.p.i. (Figure S1A–F).

**FIGURE 3:**
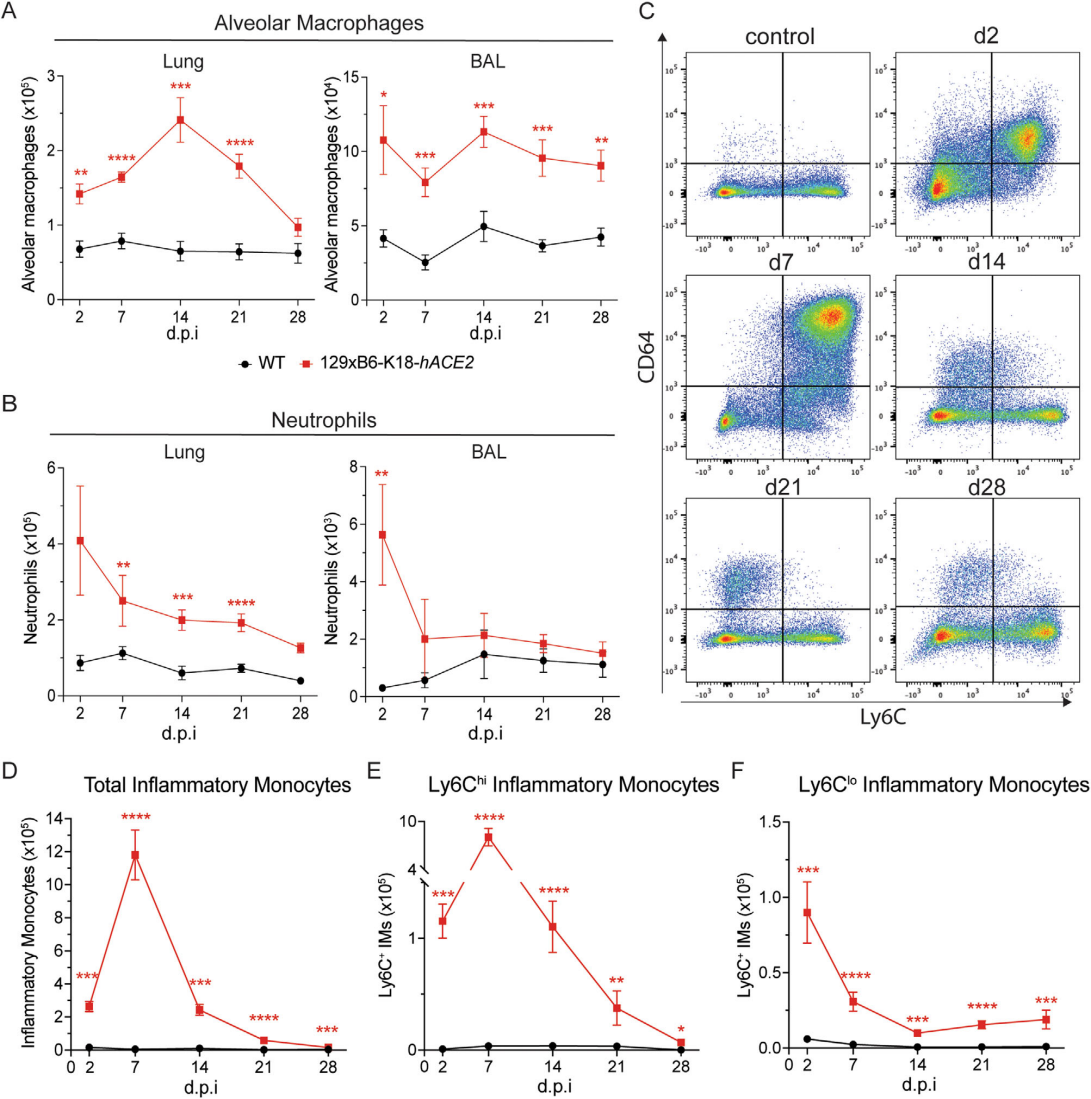
**Persistent inflammation in the airways independent of innate immune cell recruitment. (A, B)** Total cell numbers of alveolar macrophages and neutrophils in the lung and BAL at 2, 7, 14, 21, and 28 d.p.i. **(C)** Representative flow cytometry plots of lung and BAL cells gated on live, CD45^+^, Ly6G^−^, Siglec‐F^−^, CD11b^+^, CD64^+^ cells. Total cell numbers of **(D)** CD11b^+^CD64^+^ inflammatory monocytes, **(E)** Ly6C^+^, and **(F)** Ly6C^−^ populations in the lung at 2, 7, 14, 21, and 28 d.p.i. All data are shown as mean ± SEM. At 7 d.p.i., *n* = 12/group (three experiments); at 2, 14, 21, and 28 d.p.i., *n* = 8–10/group (two experiments). Statistical analysis was conducted using two‐tailed Student's *t*‐test within each time point post‐infection. *p < 0.05, **p < 0.01, ***p < 0.001, ****p < 0.0001.

Since chemokines involved in neutrophil and monocyte recruitment were upregulated, immune cell composition in the BAL and lungs of infected mice was assessed using flow cytometry (gating shown in Figure S2A). Infected 129xB6‐K18‐*hACE2* mice exhibited a marked increase in alveolar macrophages, Ly6G^+^ neutrophils, and CD11b^+^CD64^+^ inflammatory monocytes in the lungs compared with WT mice (Figure 3A–F). In the BAL, alveolar macrophages remained consistently elevated up to 28 d.p.i., while in the lungs they peaked at 14 d.p.i. (Figure 3A). Neutrophil recruitment into the airways was highest at 2 d.p.i., and while their numbers decreased over time, they remained significantly elevated in lung tissue up to 21 d.p.i. (Figure 3B).

CD11b^+^CD64^+^ inflammatory monocyte recruitment is essential in the early antiviral host response. Their recruitment was significantly increased in the lungs of 129xB6‐K18‐*hACE2* mice compared with WT, peaking at 7 d.p.i. and remaining significantly higher than WT mice up to 28 d.p.i. (Figure 3C,D). Analysis of the monocyte cell surface marker Ly6C revealed that the Ly6C^hi^ monocyte subset was the main population contributing to this increase (Figure 3C–E), while the Ly6C^lo^ monocyte population peaked at 2 d.p.i. and subsequently decreased but stayed significantly elevated throughout the time course (Figure 3F). These findings indicate robust early recruitment of innate immune cell populations into the lungs and airways of 129xB6‐K18‐*hACE2* mice upon low‐dose SARS‐CoV‐2 infection, and notably persisting well beyond resolution of acute disease.

### 2.4 Sustained Inflammation and T Cell Recruitment Upon Low‐Dose SARS‐CoV‐2 Infection

**FIGURE 4:**
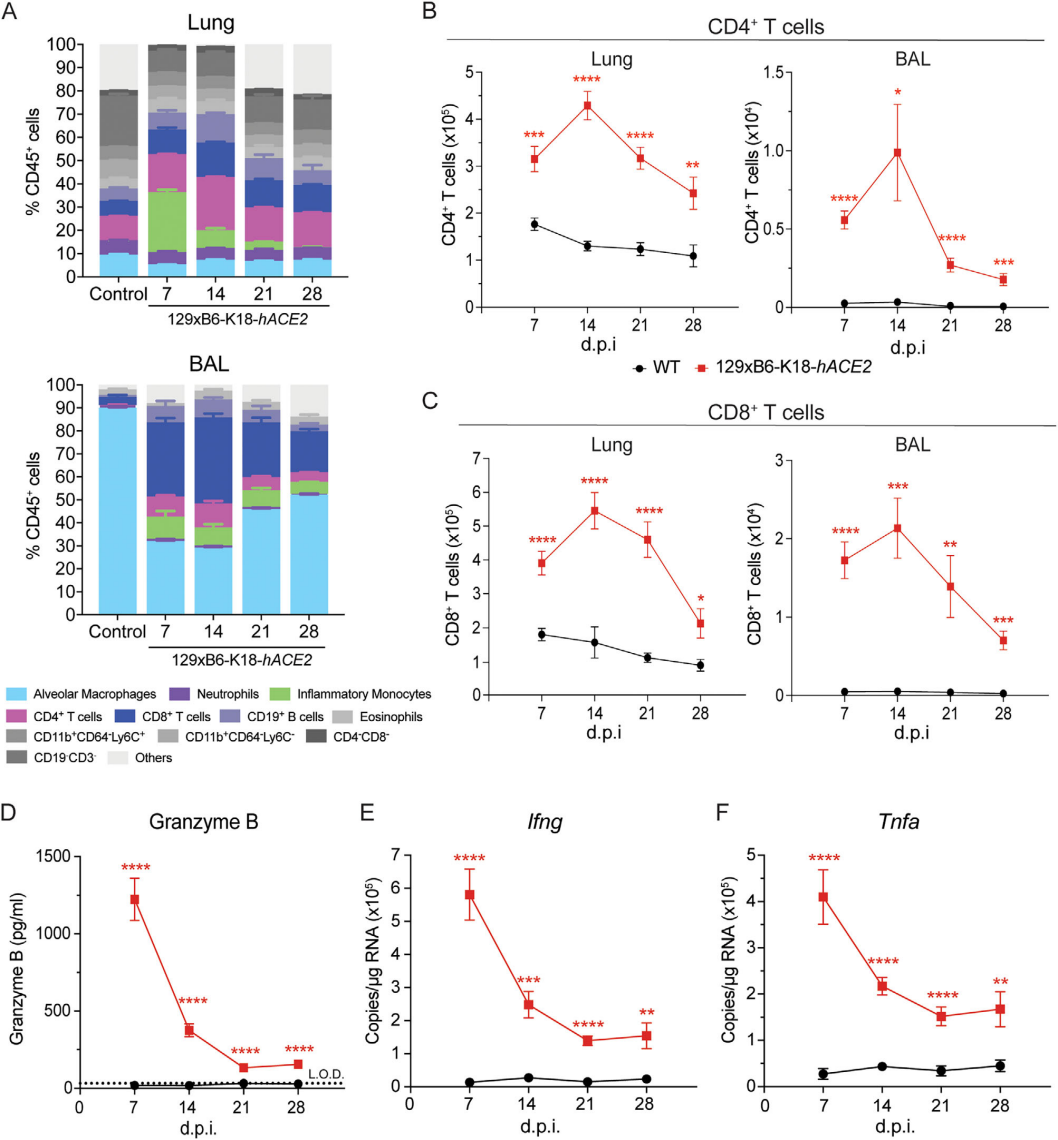
**Sustained inflammation and T cell recruitment in the airways following SARS‐CoV‐2 infection. (A)** Frequency of alveolar macrophages, neutrophils, inflammatory monocytes, CD4^+^ and CD8^+^ T cells in the lung and BAL fluid at different time points after infection of 129xB6‐K18‐*hACE2* and WT mice. Total cell numbers of **(B)** CD4^+^ and **(C)** CD8^+^ T cells in the lung and BAL at 7, 14, 21, and 28 d.p.i. **(D)** Concentration of Granzyme B in the BAL fluid measured via ELISA. Expression of immune mediators. **(E)** *Ifng*, **(F)** *Tnfa* in the lung tissue relative to *Gapdh*, measured via qRT‐PCR. L.O.D.= Limit of detection. All data are shown as mean ± SEM. At 7 d.p.i., *n* = 12/group (three experiments); at 14, 21, and 28 d.p.i., *n* = 8–10/group (two experiments). Statistical analysis was conducted using two‐tailed Student's *t*‐test within each time point post‐infection. *p < 0.05, **p < 0.01, ***p < 0.001, ****p < 0.0001.

Since the early innate immune response did not fully account for the large increase in BAL cell numbers at 14 d.p.i. (Figure 3) and sustained lung inflammation up to 28 d.p.i. (Figure 2), we next analysed the T cell compartment in infected 129xB6‐K18‐*hACE2* and WT mice (gating strategy in Figure S2B). CD4^+^ and CD8^+^ T cells constituted the majority of infiltrating immune cells in the BAL and a large proportion of CD45^+^ cells in the lungs of infected 129xB6‐K18‐*hACE2* mice (Figure 4A). CD4^+^ T cell infiltration was significantly higher in 129xB6‐K18‐*hACE2* mice than in WT mice at 7 d.p.i. in both lung and BAL, peaked at 14 d.p.i., and remained significantly elevated up to 28 d.p.i. (Figure 4B). CD8^+^ T cell infiltration followed a similar trajectory in both the lung and BAL (Figure 4C). The kinetics of both T cell populations mirrored the trends seen in the overall immune cell infiltration into the lungs and BAL (Figure 2F,G), suggesting that the persistent airway inflammation post‐viral clearance in this transgenic mouse model of low‐dose infection may be linked to T cells rather than innate immune cells.

To decipher the role of T cells in the inflammatory milieu of the airways, the expression of several T cell‐derived inflammatory mediators was assessed. *Il17* gene expression peaked at 7 d.p.i. but was not higher in 129xB6‐K18‐*hACE2* mice compared with WT mice at later time points, suggesting minimal involvement of Th17 cells contributing to the sustained inflammation (Supp. Figure 1G). Notably, Granzyme B, *Ifng*, and *Tnfa* gene expression peaked at 7 d.p.i. in the BAL and lungs, respectively, and remained significantly elevated in infected 129xB6‐K18‐*hACE2* mice until 28 d.p.i. (Figure 4D–F).

### 2.5 Prolonged CD4^+^ Effector T Cell Infiltration and Activation in 129xB6‐K18‐*hACE2* Mice

**FIGURE 5:**
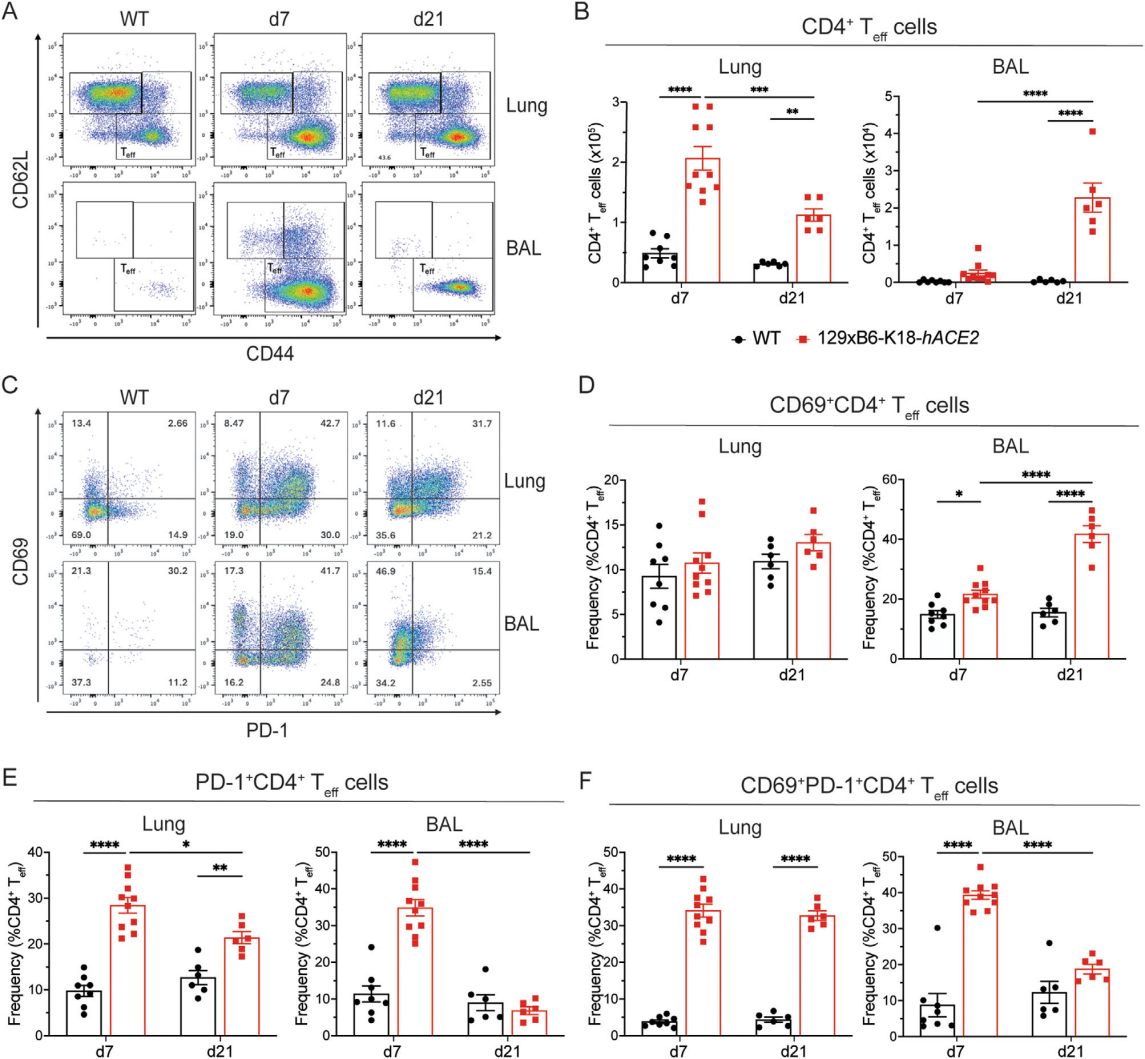
**Decreased CD4^+^ effector T cell activation at 21 days post‐infection. (A)** Representative flow cytometry plots of lung and BAL cells gated on live, CD45^+^, Ly6G^−^, Siglec‐F^−^, CD11b^−^, CD11c^−^, CD19^−^, CD3^+^, CD4^+^ cells. **(B)** Total number of CD62L^−^CD44^+^CD4^+^ effector T cells (T_eff_) in the lung and the BAL at 7 and 21 d.p.i. **(C)** Representative flow cytometry plots of lung and BAL cells gated on live, CD45^+^, Ly6G^−^, Siglec‐F^‐^, CD11b^−^, CD11c^−^, CD19^−^, CD3^+^, CD4^+^, CD62L^−^, and CD44^+^ cells. Proportion of CD62L^‐^CD44^+^CD4^+^ T_eff_ that are **(D)** CD69^+^ and **(E)** PD‐1^+^ in the lung and the BAL at 7 and 21 d.p.i. Data shown as mean ± SEM with *n* = 6–10/group (two experiments). Statistical analysis was conducted using a one‐way ANOVA with Tukey's honest significant difference test to correct for multiple comparisons. *p < 0.05, **p < 0.01, ***p < 0.001 ****p < 0.0001.

To further investigate the role of T cells during prolonged inflammation upon low‐dose SARS‐CoV‐2 infection, we identified CD4^+^ effector T (T_eff_) cells in the BAL and lungs (CD44^+^CD62L^‐^; gating in Figure S2B) at day 7 and 21 d.p.i. At 7 d.p.i. the CD44^+^CD62L^‐^CD4^+^ T_eff_ cell population was significantly larger in the lungs of infected 129xB6‐K18‐*hACE2* mice compared with WT mice, while numbers in the BAL remained at baseline. By 21 d.p.i., lung CD4^+^ T_eff_ cell numbers had decreased, albeit still higher than baseline, and a robust CD4^+^ T_eff_ cell population was present in the BAL of 129xB6‐K18‐*hACE2* mice (Figure 5A,B). Examination of early T cell activation markers CD69 and PD‐1 [28, 29] within the CD4^+^ T_eff_ cell population in the lungs revealed no difference in the frequency of CD69 single‐positive cells but the proportion of PD‐1 single‐positive CD4^+^ T_eff_ cells were increased at 7 d.p.i and decreased over time, although these levels remained significantly elevated at 21 d.p.i. in 129xB6‐K18‐*hACE2* compared with WT mice (Figure 5C,E). In addition, approximately 40% of lung CD4^+^ T_eff_ cells in 129xB6‐K18‐*hACE2* mice expressed both CD69 and PD‐1 and remained significantly elevated at 21 d.p.i. (Figure 5C–F).

The CD4^+^ T cell dynamics in the BAL were particularly revealing. Although CD4^+^ T_eff_ cell numbers were comparable between 129xB6‐K18‐*hACE2* and WT mice at 7 d.p.i., a significantly greater proportion of these cells expressed CD69, PD‐1, or both in the 129xB6‐K18‐*hACE2* mice, indicating increased activation (Figure 5C–F). In infected 129xB6‐K18‐*hACE2* mice, CD69 single‐positive CD4^+^ T_eff_ cell frequency increased over time, whereas the proportion of PD‐1 expressing cells (single‐ or double‐positive) decreased to baseline levels, suggesting a temporal PD‐1 downregulation (Figure 5C–F). Thus, despite unchanged cell numbers at 7 d.p.i., a significant subset of CD4^+^ T_eff_ cells in the BAL of infected 129xB6‐K18‐*hACE2* mice was activated. Overall, sustained CD4^+^ T_eff_ cell numbers in both lungs and BAL indicate prolonged inflammation post‐viral clearance and weight recovery.

### 2.6 Persistence of CD69^+^CD8^+^ T_eff_ Cells at 21 Days Post‐SARS‐CoV‐2 Infection

**FIGURE 6:**
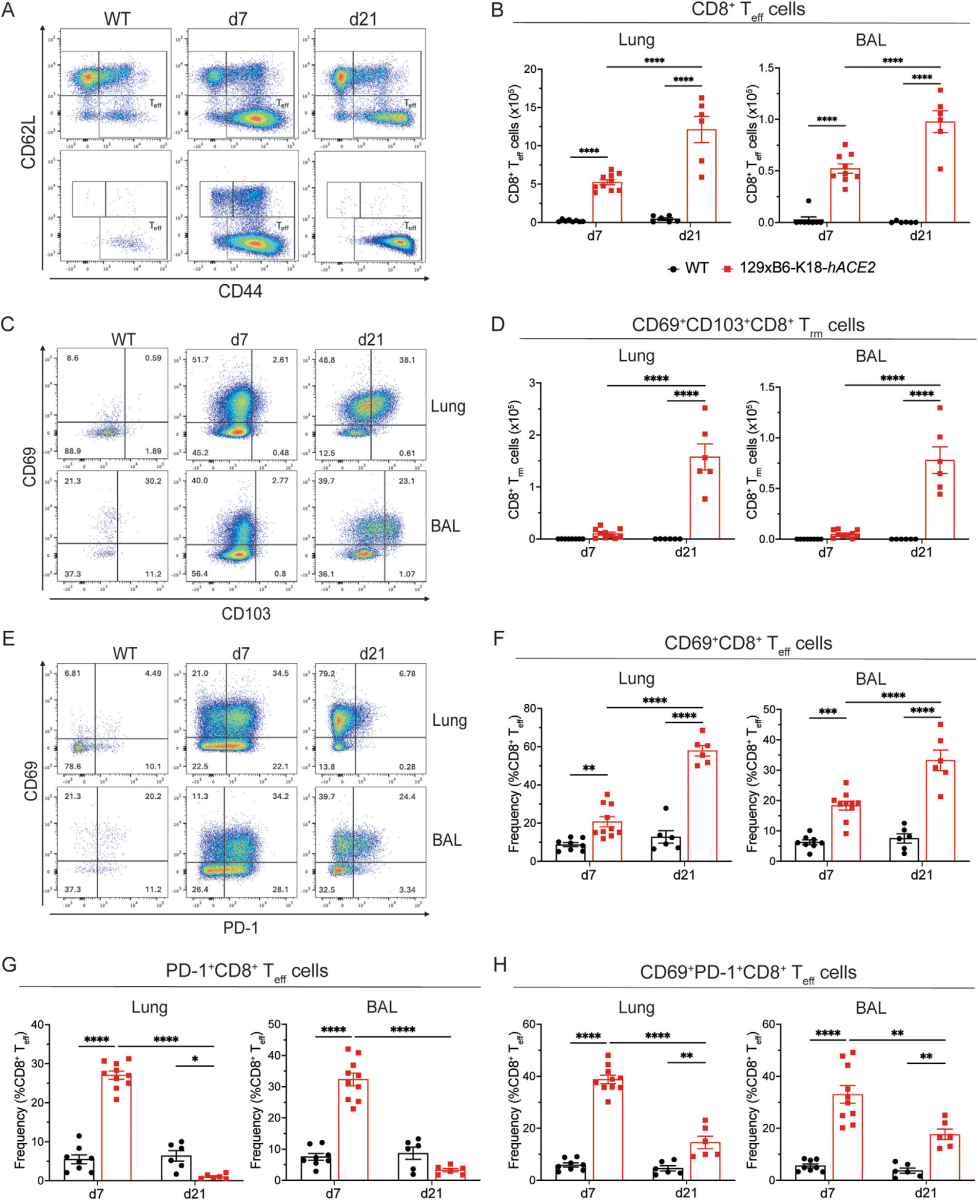
**Increased CD8^+^ effector T cell activation at 21 days post‐infection**. **(A)** Representative flow cytometry plots of lung and BAL cells gated on live, CD45^+^, Ly6G^−^, Siglec‐F^−^, CD11b^−^, CD11c^−^, CD19^−^, CD3^+^, CD8^+^ cells. **(B)** Total number of CD62L^−^CD44^+^CD8^+^ effector T cells (T_eff_) in the lung and the BAL at 7 and 21 d.p.i. **(C)** Representative flow cytometry plots of lung and BAL cells gated on live, CD45^+^, Ly6G^−^, Siglec‐F^−^, CD11b^−^, CD11c^−^, CD19^−^, CD3^+^, CD8^+^, CD62L^−^, and CD44^+^ cells. Proportion of CD62L^−^CD44^+^CD8^+^ T_eff_ that are **(D)** CD69^+^ and **(G)** PD‐1^+^ in the lung and the BAL at 7 and 21 d.p.i. **(E)** Representative flow cytometry plots of lung and BAL cells gated on CD8^+^ T_eff_ cells. **(F)** Proportion of CD69^+^CD103^+^CD8^+^ resident memory T cells (T_rm_) in the lung and the BAL at 7 and 21 d.p.i. Data shown as mean ± SEM, *n* = 6–10/group (two experiments). Statistical analysis was conducted using a one‐way ANOVA with Tukey's honest significant difference test to correct for multiple comparisons. *p < 0.05, **p < 0.01, ***p < 0.001, ****p < 0.0001.

Having identified sustained presence and activation of CD4^+^ T_eff_ cells in the lungs and BAL of 129xB6‐K18‐*hACE2* mice upon low‐dose SARS‐CoV‐2 infection, the dynamics of CD8^+^ T_eff_ cells (CD44^+^CD62L^−^; gating in Figure S2B) were investigated. CD8^+^ T_eff_ cells in both the lung and BAL were significantly higher in infected 129xB6‐K18‐*hACE2* mice, compared with WT mice, and notably increased over time (Figure 6A,B).

CD103 is an integrin that binds E‐cadherin on epithelial cells and promotes tissue retention [30]. Co‐expression of CD103 and CD69 on CD8^+^ T_eff_ cells identify tissue resident memory CD8^+^ T cells (T_rm_) [30]. In our mouse model, a distinct CD8^+^ T_rm_ cell population emerged in the lung and BAL of 129xB6‐K18‐*hACE2* mice by 21 d.p.i. (Figure 6C,D).

We next examined CD8^+^ T_eff_ cell activation via the expression of CD69 and PD‐1 on CD103^‐^CD8^+^ T_eff_ cells. The proportion of CD69 single‐positive CD8^+^ T_eff_ cells was significantly increased at 7 d.p.i. in infected 129xB6‐K18‐*hACE2* mice, compared with WT mice, and increased over time in both the lungs and BAL (Figure 6E,F). In contrast, the proportion of PD‐1 single‐positive CD8^+^ T_eff_ cells was significantly elevated in 129xB6‐K18‐*hACE2* mice at 7 d.p.i., but decreased over time in both lung and BAL to below baseline levels by 21 d.p.i. (Figure 6E–G). Similarly, CD69^+^PD‐1^+^CD8^+^ T_eff_ cells were significantly elevated at 7 d.p.i. and decreased over time, but remained higher than WT mice at 21 d.p.i. in both lung and BAL (Figure 6E–H). Overall, these data show increasing recruitment and activation (via CD69^+^) of CD8^+^ T_eff_ cells until 21 d.p.i., long after viral clearance and weight recovery.

### 2.7 Discussion

Long COVID and persistent post‐acute sequelae of SARS‐CoV‐2 affect over 400 million individuals globally, arising despite resolution of acute infection and viral clearance [31]. This complex, disabling multi‐system disorder lacks well‐characterised animal models to study its immunological underpinnings. To study prolonged lung inflammation after SARS‐CoV‐2 infection, we used a genetic cross of 129S1xC57BL/6‐K18‐*hACE2* mice [25] inoculated with low‐dose ancestral SARS‐CoV‐2 (5 × 10^2^ PFU). After infection, the mice showed weight loss and viral load, but recovered and cleared the virus by 14 d.p.i. Unlike models with early mortality, these mice survived, enabling investigation of persistent lung and airway (BAL) inflammation, which peaked at 14 d.p.i. and persisted to 28 d.p.i.

The 129xB6‐K18‐*hACE2* model was originally developed by Robertson et al. [25] by crossing diverse founder strains with *hACE2* transgenic mice [25]. Using 10^3^ PFU ancestral SARS‐CoV‐2, they observed strain‐dependent responses: complete lethality in CAST and WSB crosses with sex‐dependent sensitivity to infection; 20%–30% survival in C57BL/6‐K18‐*hACE2* mice; 80%–90% survival without weight loss in PWKxC57BL/6‐K18‐*hACE2* mice; and 80% survival and 10%–15% weight loss in 129xB6‐K18‐*hACE2* mice [25, 32]. Consistent with Robertson et al. [25], in our study, 129xB6‐K18‐*hACE2* mice infected with 5 × 10^2^ PFU SARS‐CoV‐2 showed similar weight loss trajectories, peaking at 15% on 7 d.p.i.

A significant subset of SARS‐CoV‐2‐infected patients show persistent health problems after recovery [33, 34] and sustained inflammation [35, 36]. Several mouse studies have attempted to investigate prolonged inflammation and long‐term effects postinfection. Choi et al. [37] utilised a humanised ACE2 mouse model (*hACE2* ^KI^) to study effects up to 21 d.p.i., but reported no weight loss or pulmonary immune infiltration, instead observing tau accumulation in the brain. K18‐*hACE2* mice are widely used to model severe infection and test vaccines; however, lower‐dose infections aimed at extending survival for later timepoint studies have yielded inconsistent outcomes. For instance, 2 × 10^4^ PFU and 10^3^ TCID_50_ SARS‐CoV‐2 doses reduced fatality to 50% in some studies [23, 24]. A dose of 2 × 10^2^ PFU resulted in 40% fatality by 11 d.p.i. in one study [22], but only mild disease in another [19]. Fumagalli et al. [38] used aerosolised 10^5^ TCID_50_ SARS‐CoV‐2 in K18‐*hACE2* mice to limit fatal neuroinvasion and proposed it as a model for long‐term infection. However, only weight loss and clinical score were assessed (20 d.p.i.) [38]. Further studies are needed to understand viral clearance and inflammation. Sefik et al. [39] developed a humanised chronic infection model using adeno‐associated virus (AAV) delivery of *hACE2* into MISTRG6 mice. While this model recapitulates human immune responses to severe SARS‐CoV‐2 infection up to 28 d.p.i. and detectable viral titres up to 35 d.p.i., it introduces variables such as insufficient lung coverage by transduction or irradiation‐related side effects. In contrast, the low‐dose infection model in 129xB6 K18‐*hACE2* mice used in this study is genetically stable, supports survival, and enables investigation of prolonged lung inflammation post‐infection with fewer confounding factors.

In our model, neutrophils peaked early at 2 d.p.i., unlike in the humanised MISTRG6 model, where neutrophil recruitment was absent [39]. Monocytes peaked at 7 d.p.i., later than 4 d.p.i. in the MISTRG6 model, and remained significantly elevated until 28 d.p.i., and macrophage numbers were consistently high throughout, mirroring sustained monocyte‐derived macrophage levels observed in severe COVID‐19 patients [27, 40]. We further observed elevated amphiregulin and IL‐33 gene expression in the lungs, mirroring trends observed in long COVID patients [41, 42]. Infected alveolar epithelial cells are thought to be the primary source of these mediators, exacerbating pulmonary inflammation and immune dysfunction [41, 42], and sustained amphiregulin activation may contribute to lung fibrosis and pulmonary symptoms of persistent lung inflammation [43].

Sustained IFN‐γ responses have been reported in human SARS‐CoV‐2 infection [27, 28, 29, 30, 31, 32, 33, 34, 35, 36, 37, 38, 39, 40, 41, 42, 43, 44], with activated pulmonary T cells as the primary source [39]. We similarly report sustained IFN‐γ in the BAL, along with elevated CD4^+^ and CD8^+^ T cell numbers up to 28 d.p.i. in the lung and airways. While early T cell responses correlate with viral control and mild disease [45], elevated T cell levels post‐viral clearance have been shown to correlate with persistent systemic inflammation and long COVID symptoms [7]. Furthermore, recent evidence suggests that persistent CD8^+^ T cells may impair alveolar epithelial cell regeneration via macrophage activation [46]. The persistent activation of T_eff_ cells aligns with studies of patients experiencing prolonged pulmonary symptoms, where activated T cells can persist for months following acute infection [47]. While we observed PD‐1 downregulation in CD8^+^ T_eff_ cells, most human studies report PD‐1 upregulation post‐viral clearance, suggesting T cell exhaustion [7]. Additional markers such as CD25 and Ki67 would help clarify the dynamics of T cell activation in our model. However, the sustained presence of T_eff_ cells suggested ongoing immune activation, which may lead to functional impairment where T cells remain activated but fail to control infection or resolve inflammation. This resembles T cell exhaustion in chronic viral infections, where cells express activation markers but lack effector function [48]. Such prolonged inflammation despite viral clearance underpins the chronic nature of long COVID, even after mild initial infections.

The mouse model described here offers a robust, clinically relevant tool for studying long‐term inflammation post‐viral clearance. It may also help investigate how persistent SARS‐CoV‐2‐induced inflammation affects secondary infections such as respiratory syncytial virus (RSV), where we recently reported exacerbated disease upon pre‐existing increased expression of IL‐1α and TNF‐α [49]. While not a direct model of long COVID, it recapitulates key features of post‐viral lung inflammation, including sustained recruitment of inflammatory monocytes and persistent T cell activation. Understanding the mechanisms behind persistent lung inflammation could inform therapies to mitigate long‐term respiratory complications. A specific focus on T cell inflammation may yield insights into SARS‐CoV‐2 reinfections and guide the targeted interventions toward specific T cell‐mediated inflammatory pathways.

### 2.8 Data Limitations and Perspective

Several limitations should be considered when interpreting the data from this study. First, 129xB6‐K18‐hACE2 mice exhibit non‐physiological overexpression of human ACE2, a known limitation of K18‐*hACE2* models. However, the use of heterozygous F1 129xB6‐K18‐hACE2 mice [25] may have contributed to the milder disease observed. Additionally, data collection was limited to 28 d.p.i. While later time points will be informative, our study provides detailed immune profiling up to 28 d.p.i., a timeframe that exceeds most K18‐*hACE2* models, and includes analysis beyond recovery from weight loss and viral clearance. Our low‐dose SARS‐CoV‐2 infection model induces sublethal disease with recovery and persistent lung inflammation, making it a useful platform for studying long‐term immune responses. For example, in this study, the virus specificity of persistent T cells was not assessed, and in future studies, following SARS‐CoV‐2‐specific T cells would be informative, as these have been reported in convalescent patients [50]. Although we do not model long COVID in its full complexity, it introduces a tractable system for studying post‐viral lung inflammation. We did not assess viral load in the brain, as this has been addressed previously using higher doses [25]. However, future work could determine if there is any viral persistence in the brains of surviving mice at low to intermediate doses. Lastly, fibrosis was beyond our study's scope but remains important for future exploration, including identifying sources of IL‐33, amphiregulin, other profibrotic mediators, and the roles of dendritic cells, basophils, mast cells, ILCs, and fibroblasts. More detailed analysis of lung cellular distribution is also warranted.

## 3 Materials and Methods

### 3.1 Biosafety and Ethics

All experiments were approved by the local genetic manipulation safety committee of Imperial College London, St Mary's Campus (Centre number GM77), and the Health and Safety Executive of the United Kingdom, under reference CBA1.77.20.1.

### 3.2 Mice

The original K18‐*hACE2* transgenic mouse strain is on a C57BL/6J background. F1 cross of 129S1xK18‐*hACE*2 mice (herein called 129xB6‐K18‐*hACE2)* or F1 cross of 129S1xC57BL/6J without the K18‐*hACE2* transgene (hereby referred to as WT) were generated at The Jackson Laboratory (129S1/SvImJxB6.Cg‐Tg(K18‐ACE2)2Prlmn/J)F1/J (035934)) and shipped to Imperial College London. Experiments were conducted at Imperial College London, where all infectious work was performed in designated biosafety level 3 workspaces.

### 3.3 Virus and Infections

First wave SARS‐CoV‐2 (D614G, isolate of hCoV‐19/England/IC19/2020) was grown in African green monkey kidney cells overexpressing human ACE2 and TMPRSS2 (Vero‐ACE2‐TMPRSS2; VAT cells) [51]. For infection, mice were lightly anesthetised and instilled intranasally (i.n.) with different doses of SARS‐CoV‐2 or PBS in 100 µl. Mice were monitored daily for signs of disease (fur, posture, activity) and weight loss, and symptomatic infection was based on weight loss >5%.

### 3.4 Plaque Assays

SARS‐CoV‐2 titer was assessed in lungs at 2, 7, 10, 14, 21, and 28 d.p.i. using a plaque assay as described previously [17].

### 3.5 BAL Cell Processing

Bronchoalveolar lavage (BAL) was collected by flushing the lungs three times with 1 ml PBS supplemented with 5 mM EDTA (Life Technologies). BAL cells and supernatant were separated by centrifugation, and BAL supernatants were exposed to UV light for 2 min to inactivate SARS‐CoV‐2. Red blood cells were lysed in the BAL cells using ammonium–chloride–potassium (ACK) buffer.

### 3.6 Isolation of Lung Cells

Mice were sacrificed at 2, 4, 7, 10, 14, 21, and 28 d.p.i., and lungs were perfused with PBS. To obtain lung leukocytes, lung lobes were cut into smaller pieces and incubated in complete DMEM (cDMEM, supplemented with 10% fetal bovine serum, 2 mM L‐glutamine, 100U/ml penicillin, and 100 µg/ml streptomycin), 1 mg/ml Collagenase D (Roche) and 30 µg/ml DNase I (Invitrogen) for 1 h at 37°C and then mashed through a 100 µm filter (BD). Red blood cells were lysed using ACK buffer.

### 3.7 RNA Isolation and Quantitative RT‐PCR

Lung tissue was homogenised in TRIzol, and RNA extraction was performed according to the manufacturer's instructions. After the chloroform step, the aqueous phase containing RNA was further processed using the RNeasy Mini Kit (QIAGEN). RNA concentration was quantified using the NanoDrop (Thermo Scientific). Two micrograms of RNA was reverse transcribed using a High‐Capacity RNA‐to‐cDNA kit (Applied Biosystems). To quantify mRNA levels in lung tissue, quantitative RT‐PCR reactions for SARS‐CoV‐2 nucleocapsid phosphoprotein *N* gene, envelope protein *E* gene, *Areg*, *Il33, Tnfa, Ifng, Il6, Il10, Il17, Cxcl1, Cxcl10, Ccl2*, and *Gapdh* were performed using primers and probes as previously described [52]. Analysis was performed using the QuantiTect Probe PCR Master Mix (QIAGEN) and the 7500 Fast Real‐Time PCR System (Applied Biosystems). For absolute quantification of *Ifng* and *Tnfa*, the exact number of copies of the gene of interest was calculated using a plasmid DNA standard curve, and the results were normalised to levels of *Gapdh* (Applied Biosystems). For relative quantification, the expression of *Areg*, *Il33, Il6, Il10, Il17, Cxcl1, Cxcl10, Ccl2* (all Applied Biosystems), and SARS‐CoV‐2 *N* and *E* gene was expressed relatively to the expression of *Gapdh*. First, the ΔCT (CT = cycle threshold) between the target gene and *Gapdh* was calculated for each sample, followed by calculation of 2^−ΔCT^. Analysis was performed using 7500 Fast System SDS Software (Applied Biosystems).

### 3.8 Flow Cytometry

After red blood cell lysis, lung and BAL cells were incubated for 30 min with fixable live‐dead aqua dye (Invitrogen), followed by fixation for 30 min with 4% paraformaldehyde (PFA) to inactivate the virus. Cells were then incubated for 20 min with a purified rat IgG2b anti‐mouse CD16/CD32 receptor antibody (BD) to block Fc binding, followed by staining with fluorochrome‐conjugated antibodies as listed in Table S1 in PBS containing 1% BSA and 5 mM EDTA for 25 min at 4°C. Samples were acquired using the BD LSR Fortessa flow cytometer (BD Bioscience) and FACSDiva software, where 250,000 single, live, CD45^+^ cells were acquired per sample. The data were further analysed on the FlowJo software (BD Bioscience).

### 3.9 ELISA

Granzyme B levels within the BAL fluid were quantified using the mouse DuoSet ELISA (R&D Systems) according to the manufacturer's instructions. IFN‐α levels were quantified following a previously established protocol [53]. Data were acquired using the FLUOstar OMEGA plate reader (BMG Labtech) at an absorbance of 450 nm and analysed using the Omega/MARS data analysis software (BMG Labtech).

### 3.10 Histology

Lungs were inflated with 1 ml of PBS, fixed in 10% formalin (Sigma‐Aldrich) for 16 h, and embedded in paraffin blocks. Sections of 4 mm were stained for H&E according to standard procedures. Sections were scanned using the Axio Scan Z1 slide scanner (Zeiss, Germany) and analysed using the Zen Blue software (Zeiss, Germany).

### 3.11 Statistical Analysis

Statistical analysis was performed using Prism 10 (Graph‐Pad Software). Data were tested for normality using the Shapiro–Wilk test. One‐way ANOVA with Tukey's post hoc test was used to compare multiple groups, while data with two groups were tested by either Student's *t*‐test or Mann–Whitney *U*‐test. As mice were sacrificed at each time point and repeated sampling was not feasible, Student's *t*‐test was used to compare 129S1xB6‐K18‐hACE2 mice to 129S1xC57BL/6J mice at each time point after infection. Log‐rank Mantel–Cox test was applied for Kaplan–Meier curve, and data are expressed as mean ± SEM, and for all tests a value of *p* < 0.05 was considered significant; *p < 0.05, **p < 0.01, ***p < 0.005, ****p < 0.001.

## Author Contributions

Sophie Y. Guan and Patricia P. Ogger designed and carried out the majority of experiments, data analysis, and visualisation. Ana Farias, Minerva Garcia Martín, and Joy Nakawesi carried out specific experiments and reviewed the paper. Nadia Rosenthal, Candice Baker, and Olivia Bedard generated and provided the 129xB6 K18‐*hACE2* mice and reviewed the paper. Cecilia Johansson supervised the project and designed the experiments. Sophie Y. Guan, Patricia P. Ogger, and Cecilia Johansson wrote the manuscript.

## Conflicts of Interest

Nadia Rosenthal, Olivia Bedard, and Candice Baker are employees of The Jackson Laboratory, where the 129xB6 K18‐*hACE2* cross was developed. The remaining authors declare no conflicts of interest.

## Peer Review

The peer review history for this article is available at https://publons.com/publon/10.1002/eji.70043.

## Ethics Statement

All animal studies were reviewed and approved by the Animal Welfare and Ethical Review Board (AWERB) at Imperial College London and approved by the UK Home Office in accordance with the Animals Act 1986 (Scientific Procedures) and ARRIVE guidelines.

## Supporting information




**Supplementary File** 1: eji70043‐sup‐0001‐SuppMat.pdf

## Data Availability

The data that support the findings of this study are available from the corresponding author upon reasonable request.
