# Supplementary material for: Sustained Lung Inflammation Post‐SARS‐CoV‐2 Infection in Mice Is Associated with Increased Pulmonary T Cells
Source: Eur J Immunol. 2025 Aug 24;55(8):e70043. doi: 10.1002/eji.70043 (PMC12375910; doi:10.1002/eji.70043)
Supplement: Supplementary file 1 — eji70043‐sup‐0001‐SuppMat.pdf [file EJI-55-e70043-s001.pdf]

Supplementary Figures

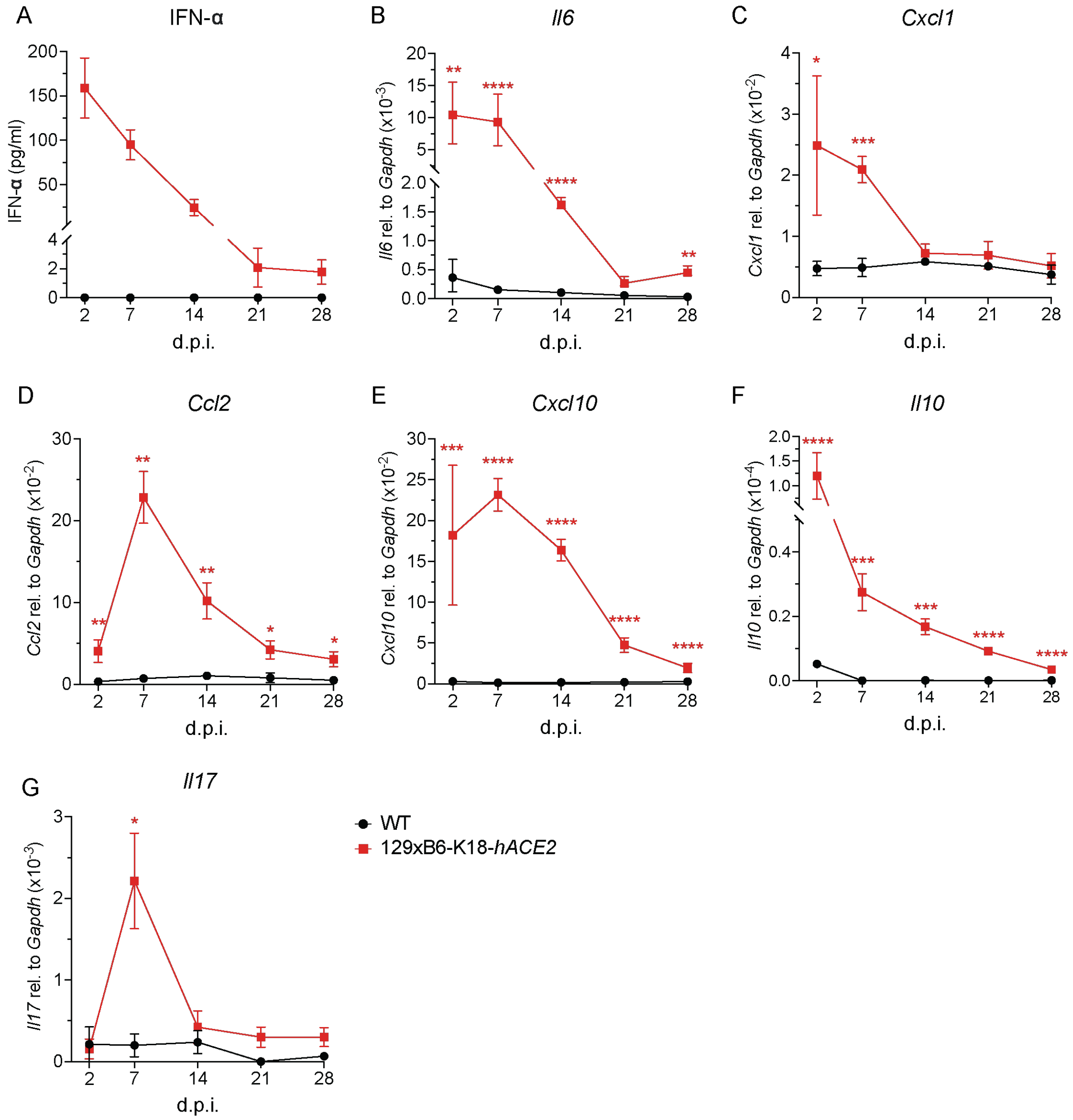

Supplementary Figure 1: Cytokine response within lungs of mice upon SARS-CoV-2 infection.

(A) Concentration of IFN-α in the BAL fluid measured via ELISA. Expression of immune mediators **(B)** *Il6*, **(C)** *Cxcl1*, **(D)** *Ccl2*, **(E)** *Cxcl10*, **(F)** *Il10*, and **(G)** *Il17* in the lung tissue relative to *Gapdh*, measured via qRT-PCR. All data are shown as mean±SEM. At 2 d.p.i., n=8-9/group, pooled from 2 experiments; at 7 d.p.i., n=12/group, pooled from 3 experiments; at 14, 21, and 28 d.p.i., n=8-10/group, pooled from 2 experiments. Data were tested for normality using the Shapiro–Wilk test. Group comparisons were performed using the Mann–Whitney U test (B, C, F) or unpaired Student's *t* test (A, D, E, G), as appropriate, within each time point. *$p < 0.05$, **$p < 0.01$, ***$p < 0.001$ ****$p < 0.0001$

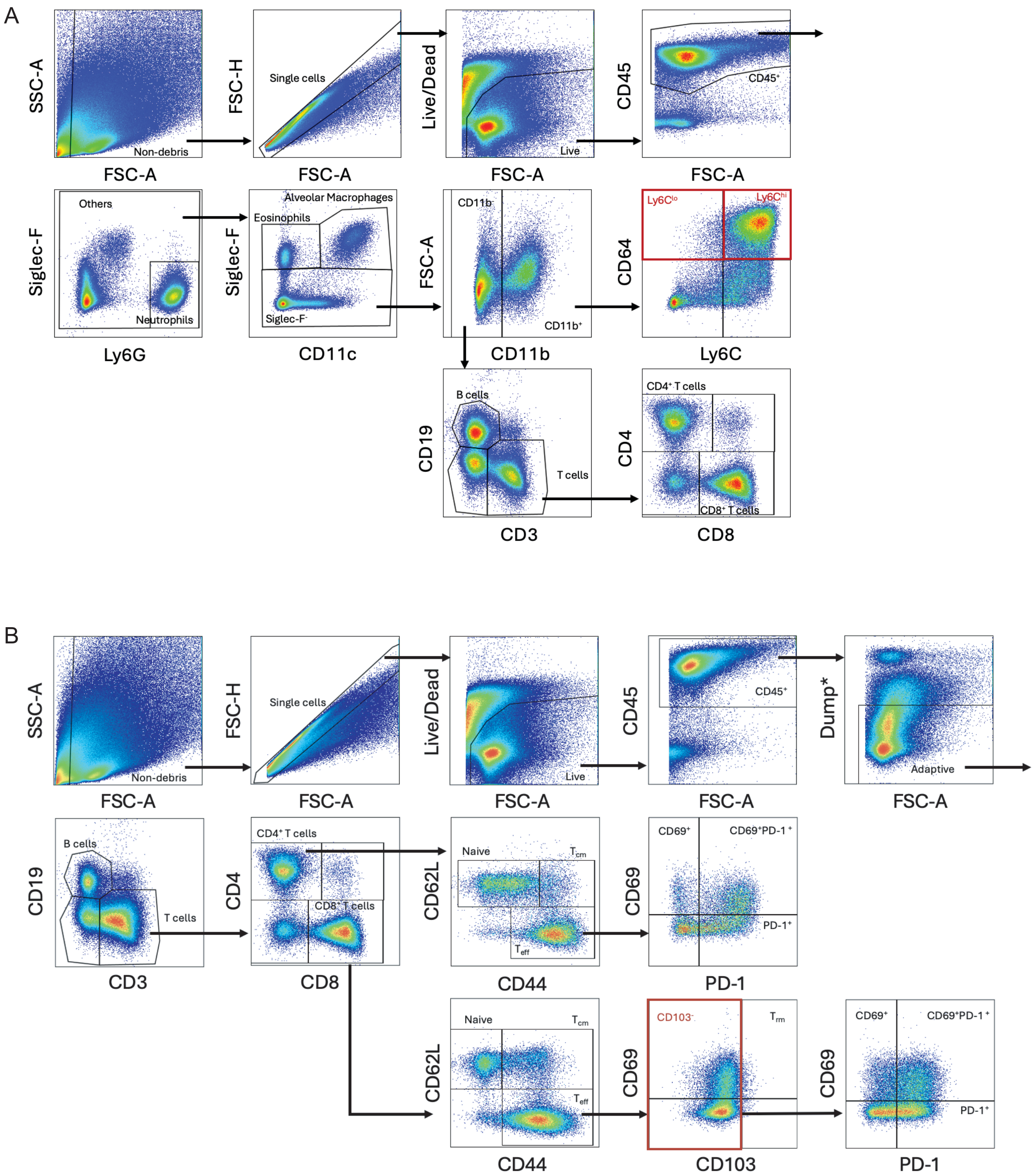

Supplementary Figure 2: Flow cytometry gating strategies.

(A) Representative gating strategy to detect innate and adaptive immune cell populations in the lung and BAL following SARS-CoV-2 infection at days 2, 7, 14, 21, and 28. Populations of interest are neutrophils, alveolar macrophages, Ly6C^{+}CD11b^{+}CD64^{+} and Ly6C^{-}CD11b^{+}CD64^{+} inflammatory monocytes (Ly6C^{hi} and Ly6C^{lo} populations indicated in red), CD4^{+} and CD8^{+} T cells. **(B)** Representative gating strategy to detect T cell populations and activation in the lung and BAL at 7 and 21 d.p.i.. Populations of interest are CD62L^{-}CD44^{+}CD4^{+} effector T cells (T$_{eff}$), CD62L^{+}CD44^{+}CD8^{+} effector T cells (T$_{eff}$), CD69^{+}CD103^{+}CD8^{+} resident memory T cells (T$_{rm}$). Additionally, expression of activation markers CD69 and PD-1 were further assessed within the CD8^{+}CD103^{-} T$_{eff}$ cells (in red). *Dump channel represents cells innate immune cell markers in APC (Siglec-F, CD11c, CD11b, Ly6G, Ly6C). Plots shown are representative of lung cells obtained from 129xB6-K18-*hACE2* mice at 7 d.p.i..

Supplementary Table 1: Antibodies used for flow cytometry

**Antibody targeting:**	**Clone**	**Fluorophore**
CD45	30-F11	PerCP-Cy5.5
CD45	30-F11	BUV395
Siglec-F	E50-2440	BV786
Siglec-F	S17007L	APC
Ly6G	1A8	AF488
Ly6G	1A8	APC
CD11c	HL3	PE-CF594
CD11c	HL3	APC
CD11b	M1/70	PE-Cy7
CD11b	M1/70	APC
CD64	X54-5/7.1	PE
LY6C	HK1.4	BV421
CD19	6D5	APC
CD19	6D5	FITC
CD3	17A2	AF700
CD3	17A2	AF700
CD4	GK1.5	BUV395
CD4	GK1.5	BV786
CD8a	53-6.7	APC-eFluor780
CD62L	MEL-14	BV650
CD44	IM7	PE-Cy7
CD69	H1.2F3	PE
CD103	M290	BUV373
PD-1	29F.1A12	BV605
